# Loneliness and Neuropsychiatric Symptoms in Cognitively Impaired Older Adults during COVID-19 Pandemic

**DOI:** 10.1093/geroni/igab046.2741

**Published:** 2021-12-17

**Authors:** Prasad Padala, Kalpana Padala, Ashlyn Jendro, Clinton Gauss, Kerrie Wilson, Aparna Das, Samuel House, Scott Mooney

**Affiliations:** 1 Central Arkansas Veterans Healthcare System, North Little Rock, Arkansas, United States; 2 Central Arkansas Veterans Healthcare System, Central Arkansas Veterans Healthcare System, North Little Rock, Arkansas, United States; 3 University of Arkansas for Medical Sciences, Little Rock, Arkansas, United States

## Abstract

**Background:**

Cognitively impaired older adults living in the community have been vulnerable to the effects of COVID-19 confinement. The current study’s objectives were to examine the prevalence of loneliness in such adults along with impact of COVID-19 on neuropsychiatric symptoms and functional status.

**Methods:**

A cross-sectional study was conducted in community dwelling cognitively impaired older Veterans (N=41). Demographic data such as age, gender, race, and rurality were collected. Loneliness data were collected with the 3-item Loneliness Questionnaire. Cognition was assessed with the Tele-Montreal Cognitive Assessment (T-MoCA) and functional status of instrumental activities of daily living was assessed with the Functional Activities Questionnaire (FAQ). Neuropsychiatry symptoms including severity and distress were collected using the Neuropsychiatric Inventory (NPI), and change during COVID was also recorded for each symptom.

**Results:**

Demographic characteristics included: mean age of 71.9 (±8.6) years, 95.1% male, 46.3% rural, 75.6% Caucasian, and 19.5% African American. Loneliness was prevalent in most participants (62.5%). T-MoCA and FAQ mean scores were 15.1 (±4.5) and 10.0 (±8.6), respectively. Mean NPI total severity and total distress were 8.4 (±5.9) and 11.4 (±8.5), respectively. Irritability was most frequently reported symptom (65%), followed by agitation (57.5%), anxiety (55%), depression (50%), and night-time behavior (50%). A majority of the participants reported worsening of neuropsychiatric symptoms during COVID (71.1%). Among those that reported worsening neuropsychiatric symptoms, 70.4% noted an increase in ≥ two symptoms.

**Conclusion:**

Older adults with pre-existent cognitive impairment may be at high risk for loneliness and worsening of neuropsychiatric symptoms during the COVID pandemic.

